# Effects of Disinfectant Solutions Against COVID-19 on Surface Roughness, Gloss, and Color of Removable Denture Materials

**DOI:** 10.3390/jfb16120446

**Published:** 2025-11-29

**Authors:** Aikaterini Mikeli, Nick Polychronakis, Xanthippi Barmpagadaki, Gregory Polyzois, Panagiotis Lagouvardos, Spiros Zinelis

**Affiliations:** 1 Department of Prosthodontics, School of Dentistry, National and Kapodistrian University of Athens, 11527 Athens, Greece; akatmikeli@dent.uoa.gr (A.M.); nicpolis@dent.uoa.gr (N.P.); grepolyz@dent.uoa.gr (G.P.); 2 Aesthetic & Prosthesis Dental Lab, Private Practice, 18544 Pireas, Greece; xbarbagadaki@yahoo.gr; 3 Department of Operative Dentistry, School of Dentistry, National and Kapodistrian University of Athens, 11527 Athens, Greece; plagou@dent.uoa.gr; 4 Department of Biomaterials, School of Dentistry, National and Kapodistrian University of Athens, 11527 Athens, Greece

**Keywords:** disinfectants, COVID-19, roughness, gloss, color, removable dentures

## Abstract

The objective of this study was to investigate the effect of surface disinfectant solutions against COVID-19 on the surface roughness, gloss, and color of removable denture materials. Fifty rectangular metallic specimens made of Co-Cr alloy and fifty disk-shaped specimens made of PMMA were prepared according to the manufacturers’ instructions. Fifty maxillary right central incisors were also included in the study. The above-mentioned specimens were equally divided into five groups (n = 10). Four disinfectant solutions were tested (0.1 wt% NaOCl, 0.5 wt% H_2_O_2_, 78 wt% ethanol, and 1 wt% Povidone Iodine), and freshly distilled water was used as the control. To simulate clinical practice, each specimen was immersed in the disinfectant solution 15 times. All specimens were marked, and surface roughness, gloss, and color were measured before and after immersion. All roughness parameters (Sa, Sq, Sz, Sc, and Sv) and gloss values before and after immersion were statistically compared. ΔE*ab values were statistically compared with perception and acceptability thresholds according to ISO/TR 28642. No significant differences were identified for surface roughness parameters for all groups tested. All materials demonstrated a significant increase in gloss after aging regime, while only the metallic specimens illustrated ΔE*ab values higher than the acceptability threshold after disinfection with H_2_O_2_. None of the surface properties deteriorated after exposure to tested disinfectants, and thus, all of them can be effectively implemented in everyday practice.

## 1. Introduction

Since its first appearance at the end of 2019, COVID-19 has caused more than 7.1 million deaths according to World Health Organization data (Autumn 2025) [1]. The virus (SARS-CoV-2) is transmitted directly from person to person through coughing, sneezing, and saliva or indirectly via contact with surfaces or objects contaminated by the virus [2,3], and it can survive on surfaces for a period of a few hours up to a few days [4]. This means that materials used for the fabrication of removable prosthetics may become viral transmission routes since prosthetic devices are transferred from the dental laboratory to the dental office and placed in the patient’s mouth, which increases the likelihood of cross-contamination among patients and dental and laboratory personnel [5]. It is self-evident that all involved parties must implement disinfection protocols with effective solutions to prevent undesirable consequences. Sodium hypochlorite, alkaline glutaraldehyde, hydrogen peroxide, and isopropyl alcohol are the most commonly used solutions [6,7] for this purpose. However, it is unclear how the repetitive applications of these solutions may affect significant surface properties related to the esthetics and appearance of materials used for the fabrication of removable dentures. These materials are heat-cured polymethyl methacrylate (PMMA) for the fabrication of artificial teeth and denture base and cobalt-chromium (Co-Cr) alloys for the fabrication of removable partial denture frameworks. These materials have a long record in the fabrication of removable restorations since they combine adequate mechanical properties, biocompatibility, and good esthetics.

Surface roughness, gloss, and color are considered among the important properties from the standpoint of esthetics, hygiene, and patient comfort and acceptance. A smooth surface enhances esthetics, minimizes the concentration of microorganisms and bacterial plaque on its surface [8], and increases patient comfort [9]. Surface roughness depends on the material structure and the final steps of surface finishing [10]. Smoother surfaces have increased surface gloss [10], which minimize the retention of pigments and thus show increased color stability. A strong positive correlation between surface roughness and bacterial adhesion on PMMA surfaces has also been reported [11,12], while it is considered that smooth surfaces with Sa below the nominal threshold of 200 nm (0.2 μm) hinder the accumulation of microorganisms [13]. Color is a significant aspect of esthetic appearance that makes prosthetic restoration acceptable in the first place, but color changes over time indicate material aging or deterioration. Denture materials are exposed to a great number of coloring agents, including foods, beverages, cleansers, and disinfectants, all of which may affect the appearance of a prosthetic device [14]. Gloss is a vital property of surface appearance and describes the capacity of a surface to reflect direct light and depends mainly on the surface finish. If the reflecting surface is smooth, then light is reflected in a regular manner resembling the appearance of teeth. On the other hand, if the surface is rough, light is reflected in a diffused manner, and the surface appears dull and matte [15].

In general, any deterioration of surface properties of removable denture materials is not desirable as it degrades esthetics and reduces patient acceptance. In light of these findings, numerous studies have focused on the effect of various deteriorating agents (i.e., beverages, staining solutions, cleansers, disinfectants, and others) on roughness [7,16,17,18,19,20,21,22,23,24,25,26,27,28,29,30,31,32], color stability [19,22,26,33,34,35,36,37,38,39,40,41], and gloss [15,26,38,42] of denture materials. Of course, these studies do not share the same artificial aging regime and, most importantly, do not include all types of denture materials (alloy, denture base, and artificial teeth), and thus, conclusive remarks are limited. Most importantly, studies dealing with disinfectants against human coronavirus inactivation are scarce, including only dental alloys [43] or artificial acrylic teeth [44], and thus, there is room for further testing and research.

Furthermore, the question of the significance of the effect of such solutions on a removable denture by their use in the necessary clinic visits during the initial adjustment period and the subsequent follow-up by the dental practitioner during its life span has not been answered [45,46,47,48].

Therefore, despite there being numerous studies on the effect of various solutions on surface properties of removable prosthetic materials, only a few include solutions recommended for surface disinfection against COVID-19, and thus, the aim of this study was to measure the effect of currently used surface disinfectant solutions on the surface roughness, gloss, and color of denture base acrylic resin, artificial denture teeth, and Co-Cr alloys. The null hypothesis was that disinfectants against SARS-CoV-2 do not cause significant effects on roughness, color, and gloss over a specified regime of artificial aging.

## 2. Materials and Methods

### 2.1. Sample Preparation and Materials

Metallic framework (MF): For the preparation of Co-Cr specimens, 50 rectangular wax patterns (12 mm × 6 mm × 0.5 mm) were prepared (IQ sticks, Yeti Dental, Engen, Germany). These wax patterns were created using a silica-based investment material (GC Stellavest, GC Europe, Leuven, Belgium) and cast with remanium^®^ GM 380+ (Dentaurum, Ispringen, Germany), a Co-Cr alloy indicated for the fabrication of partial denture frameworks. Casting was performed on a centrifugal casting machine (Ducatron S3, Ugin’Dentaire, Seyssins, France). The firing program for the mold and the casting temperature of the Co-Cr alloy were set according to the manufacturers’ instructions. All specimens were cleaned by air abrasion using alumina oxide particles of 110 μm, with 0.3 MPa pressure, an incidence angle of 45°, and 10 mm distance followed by steam-jet treatment and air-drying. Then, they were conventionally ground and polished with dental burs and polishing instruments (Komet Dental, Gebr. Brasseler GmbH & Co. KG, Lemgo, Germany) and finally cleaned with a steam jet before allowing the specimens to dry in ambient temperature. The specimens were randomly divided into 5 groups (n = 10) corresponding to 5 immersion solutions (Table 1) and numbered.

Denture base (DB): For the preparation of PMMA specimens, 50 disk-shaped specimens (25 mm × 3 mm) were prepared employing a commercial PMMA (Vertex Rapid Simplified, Vertex Dental, Soesterberg, The Netherlands) denture base material. The specimens were prepared employing heat-curing methodology and surface grinding and polishing, as proposed by the manufacturer. The specimens were randomly divided into 5 groups (n = 10) corresponding to 5 immersion solutions and numbered.

Artificial teeth (AT): For testing denture teeth, 50 maxillary right central incisors (Vitapan, VITA Zahnfabrik, Bad Säckingen, Germany) of shade A3 were used. The teeth were divided into five groups of ten (n = 10) specimens each and numbered on their lingual surfaces.

Finally, four disinfectant solutions and freshly distilled water (used as the control) were included in this study. The solutions used were 0.1 wt% NaOCl, 0.5 wt% H_2_O_2_, 78 wt% ethanol, and 1 wt% Povidone Iodine (Table 1). The pH values of all solutions were measured by employing 200 mL of freshly made solutions using a calibrated pH meter (C1010, Consort bvba, Turnhout, Belgium). The pH meter was initially calibrated by employing two standards of 4 and 7 pH values.

### 2.2. Disinfection Protocol

To simulate clinical practice during pre-insertion, insertion, and post insertion follow up, all samples were hydrated in distilled water for 48 h before the baseline measurement (T0). Then, each specimen was immersed in the disinfectant solution for the time indicated in Table 1 and rinsed with distilled water for 1 min. The procedure was repeated 15 times to roughly simulate the minimal times of disinfection cycles required during initial insertion/adjustment (3 times), post insertion adjustment (6 times) [46], and annual recall visits up to 6 years (six times) in which removable prosthodontics restorations are commonly replaced [49]. In Table 1, the immersion regime and total exposure time is presented for each group. The control group remained in distilled water for 15 min. Surface roughness, gloss, and color were measured before (T0) and after the completion of artificial aging (T1).

### 2.3. Surface Roughness Measurements

The central part of each specimen was analyzed by means of optical profilometry employing Vertical Scanning Interferometry (VSI). One measurement was taken from each specimen. An optical profilometer (Wyko NT-1100, Tuscon, AZ, USA) with a 20× nominal magnification lens was employed, and the acquisition window was 231 μm × 303 μm. a Gaussian regression filter was applied with a 0.0025 mm short wavelength cut off filter to remove the waviness of the surface, and then the following surface roughness parameters were acquired: arithmetic mean deviation (Sa), root mean square height (Sq), maximum height of the surface (Sz), core void volume showing the volume of the surface (Sc) that could support from 10% to 80% of the bearing ratio, and the surface volume (Sv) showing the volume of the surface that could support from 80% to 100% of the bearing ratio. All acquired data were analyzed employing Vision 64, ver 5.7 software (Bruker Corporation, Tucson, AZ, USA).

### 2.4. Gloss Measurements

Gloss measurements were carried out employing a gloss meter (Novo-Curve; Rhopoint Instruments, East Sussex, UK) at a specular angle of 60° according to ASTM D523-14:2018 [50] and ISO 2813:2014 [51]. Initially, the glossmeter was calibrated by employing an appropriate standard (Novocurve High Gloss Calibration Standard) with a reference value of 93.7. Gloss was recorded before and after artificial aging, and gloss changes (ΔG) were determined for all specimens. To eliminate ambient interferences, all measurements were carried out in a shielded box.

### 2.5. Color Measurements

Color parameters (L*, a*, b*) of the specimens in the CIELAB system (57, 58) were measured using a portable colorimeter with a measurement window of 4 mm. (FRU-WR18; Shenzhen Wave Optoelectronics Technology Co., Ltd., Shenzhen, China). Overall color changes in teeth (T1) from baseline values (T0) were calculated in ΔΕ*ab units using the following formula:

ΔE*ab = [(ΔL*)^2^ + (Δa*)^2^ + (Δb*)^2^]^½^

where ΔL*, Δa*, and Δb* are the differences in L*, a*, and b* before and after artificial aging.

### 2.6. Statistical Evaluation

All roughness parameters and gloss values before and after immersion were statistically compared using the paired *t*-test. Normality of data was estimated using the Kolmogorov–Smirnov test, and in the absence of normality, the signed rank test was used instead.

ΔG for the same material was statistically analyzed among different solutions using a one-way ANOVA after normality and equality of variances (Brown–Forsythe) testing. In the absence of either normality or equal variance, the Kruskal–Wallis one-way Analysis of Variance on Ranks was used instead. Tukey’s post hoc test was used to identify significant differences among groups.

ΔE*ab values were initially checked for outliers using the Grubbs test at a = 0.05, and normality was estimated using the Kolmogorov–Smirnov test. Then, ΔE*ab values were compared using the *t*-test to find out whether they were higher than the 50:50% perceptibility threshold (PT) (ΔE*ab > 1.2) and/or the 50:50% acceptability threshold (AT) (ΔE*ab > 2.7), as imposed by ISO/TR 28642 [52]. In the case of significant differences, the three parameters (L, a, and b) were statistically compared with a paired *t*-test between T0 and T1 to identify the direction of color shifting.

Level of statistical significance for all analyses was set at 95% (α = 0.05), and all statistical analyses and illustrations were carried out using the Sigma Plot v.14 software (Systat Software Inc., San Jose, CA, USA) and OriginPro, v. 2021 (OriginLab Corporation, Northampton, MA, USA). All raw data are available by Supplementary Materials of this paper.

## 3. Results

### 3.1. Surface Roughness

**Figure 1:**
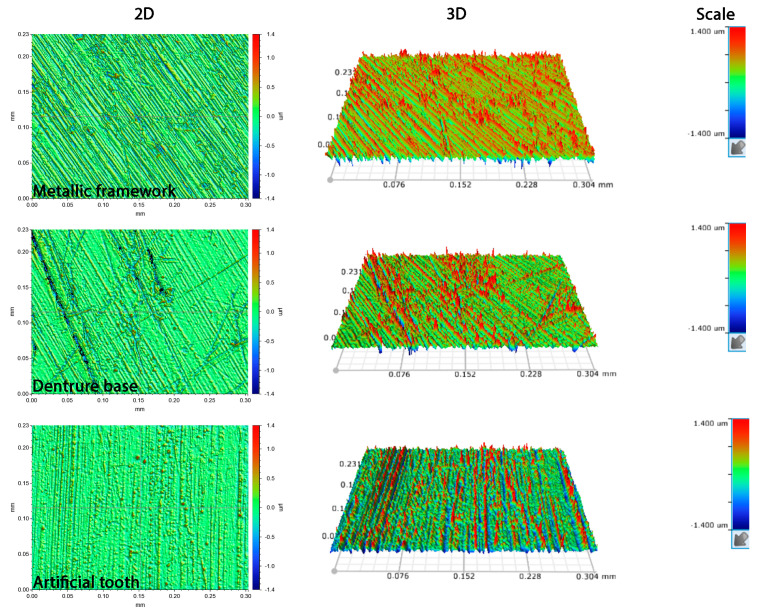
Representative 2D (**left column**) and 3D images (**central column**) before and after immersion of metallic framework, denture base, and artificial teeth surfaces. All 3D images share the same scale (**right column**). All images were taken after artificial aging in H_2_O_2_ (group HYP).

Figure 1 shows representative 2D and 3D images of all tested materials. In all cases, parallel lines are easily identified. Table 2 shows the results of surface roughness parameters tested before and after immersion cycles. No statistically significant differences were identified for all materials in all tested surface roughness parameters.

### 3.2. Gloss

Table 3 shows the gloss values of Co-Cr, denture base, and teeth specimens before and after their immersion in the solutions, along with the difference (ΔG). MF demonstrated a statistically significant increase in gloss values: the ΔG value of HYP, ETH, and PIO was significantly higher than that of WAT. The gloss values of the DB group increased significantly after immersion cycles with no statistically significant differences in ΔG values among solutions. The gloss of the AT group also increased significantly after immersion in the solutions, with the ΔG value in the SOH solution being significantly higher compared to that of the HYP, ETH, and PIO solution groups.

### 3.3. Color Measurements

**Figure 2:**
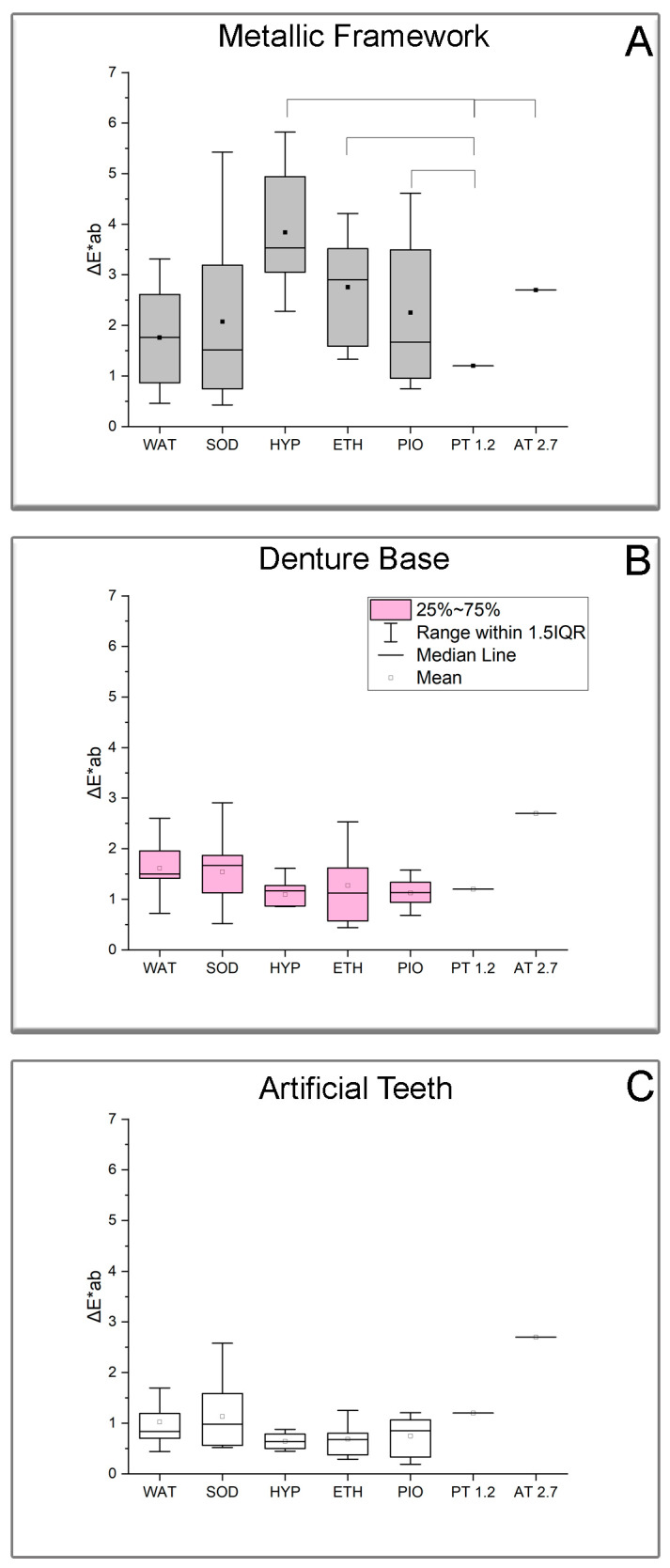
Box plot diagrams of ΔE*ab values for MF (**A**), DB (**B**), and AT (**C**). The horizontal line in the box shows the median, and the rectangular dot represents the mean. Lower and upper box lines indicate the 25% and 75% percentiles, respectively (legend is shown only in (**B**) for the sake of clarity). Horizontal lines connect groups with statistically significant differences either to PT or AT (*p* < 0.05). All diagrams share the same full-scale y axis for comparison purposes.

Figure 2 illustrates the ΔE*ab for all groups tested along with the statistical outcome. The HYP group of the metallic framework showed significantly higher ΔE*ab values compared to the PT and AT, while ETH and PIO were only higher than the PT (Figure 2A). None of the groups from the denture base and artificial teeth showed ΔE*ab values higher than PT (Figure 2B,C) or AT.

**Figure 3:**
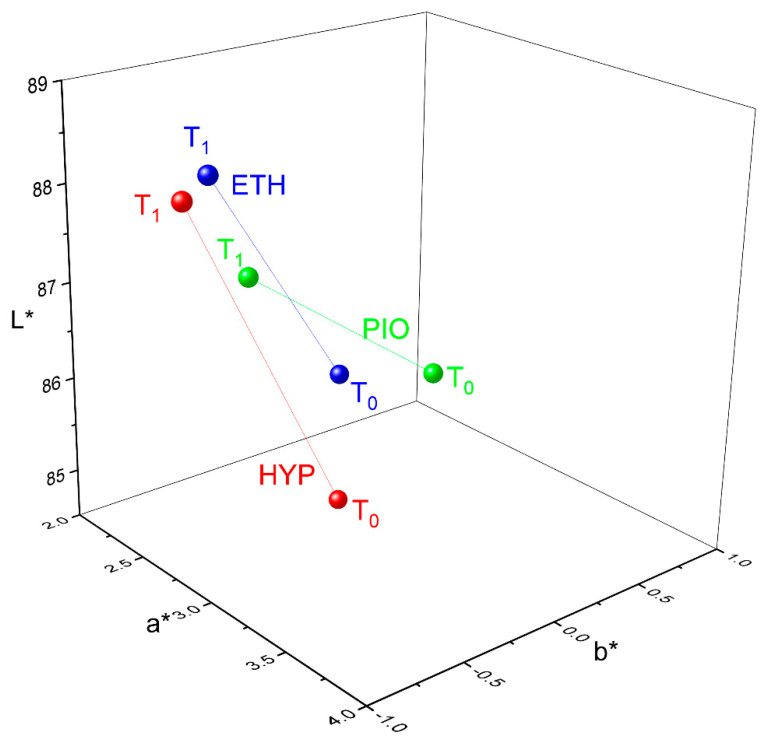
Shifting of L*, a*, and b* from T0 to T1 for HYP, ETH, and PIO groups of metallic frameworks, which demonstrated ΔE*ab values above PT and/or AT.

Figure 3 represents the shift in L*, a*, and b* parameters of the three groups of metallic frameworks, with ΔE*ab values being higher than PT and/or AT (Figure 2A). The HYP group showed statistically significant differences for L* (T0: 84.5 (1.7), T1: 88.1 (2.1) *p* < 0.001) and b* (T0: 0.0 (0.5) T1: −0.9 (0.7), *p* = 0.007). Similarly, the ETH group showed statistically significant differences for L* (T0: 85.9 (2.1), T1: 88.3 (1.8), *p* < 0.001) and b* (T0: 0.0 (0.8), T1: −0.7 (0.5), *p* < 0.001). Finally, the PIO group showed statistically significant differences only for a* (T0: 3.0 (0.1), T1: 2.6 (0.3), *p* < 0.002) and b* (T0: 0.2 (0.6), T1: −0.5 (0.3), *p* = 0.001). The differences in L* and b* imply that the HYP and ETH groups shifted to white and blue in the white–black and blue–yellow axes, respectively, while no shifting was identified in the green–red axis (a axis). In contrast, the PIO group demonstrated a shift to green and blue without shifting to the black–white axis.

## 4. Discussion

As no differences were identified in surface roughness parameters between baseline and after aging measurements, the null hypothesis should be accepted. In contrast, significant statistical differences were found for gloss and color; therefore, the corresponding null hypothesis should be rejected. Each group of material was immersed in one of the disinfectant solutions for 7.5 min or 15 min based on the period recommended for an effective disinfection against COVID-19 [6].

An optical light profilometer (non-contact) was used in this study to overcome the limitations of tactile profilometers. The former has a much higher resolution [53], while the non-contact acquisition eliminates the surface damage induced by the mechanical sensor which has been suspected of underestimating the surface roughness [54,55]. Although the majority of research papers use Ra for the quantitative characterization of roughness [13,16,17,18,27,29,31,43], the use of additional roughness parameters is recommended, as Ra only represents the arithmetic mean, and completely different surface profiles may share the same Ra [56]. Therefore, additional surface roughness parameters of amplitude, spatial, and functional groups are recommended as they are related with optical properties, bacterial adhesion fluid retention, and other properties [54,56]. In this study Sq and Sz were also determined as Sq represents an overall measure of surface roughness, although it is insensitive to peaks and valleys, while Sz represents the maximum height of the surface from the deepest valley. Two more functional parameters, Sc and Sv, are associated with the tested surfaces’ capacity of fluid retention and were also included to facilitate the explanation of the possible differences in gloss and color measurements. According to ISO 25178–2:2021 [57], the S family parameters are considered three-dimensional and thus more reliable [58]. Although a threshold for acceptable surface roughness from the standpoint of esthetics, biological complications, and longevity of restoration has not yet been set, Ra values above 200 nm (0.2 μm) have been associated with increased microorganism and plaque accumulation and a higher risk for caries and periodontal inflammation [9,13,55]. All materials tested demonstrated Sa values (Table 2) below the aforementioned threshold, and thus, their surface roughness should be considered acceptable from this standpoint.

The absence of significant differences in the surface roughness of metallic frameworks made of Co-Cr alloys are in accordance with previous findings [17] for specimens immersed in distilled water and NaOCl 0.5% for a prolonged time (1800 min). The same is true for the results of another study, where distilled water and 1.5% H_2_O_2_ solutions were used [43]. Contrary to the results of the current study, significant differences were identified after immersion in 0.5% NaOCl, a finding which should be appended to a NaOCl concentration five times higher compared to that used in this work and a prolonged immersion time (900 min) [43]. No significant differences in surface roughens (Ra) before and after immersion for the denture base material (PMMA) were reported when PMMA specimens were immersed in NaOCl 1% or water for 30 h [27] or 7 days [29], and the same was true when specimens were immersed in NaOCl 5.25% for 30 min and 7 days [16]. Only values for Ra in distilled water are available for artificial teeth, where surface roughness did not change after 1 week of immersion [30]. The results of this study fully coincide with the data in the literature showing that the surface roughness of metallic frameworks, denture base, and artificial teeth remain unaffected by the disinfectants included in this study.

Gloss meters measure the amount of light that is reflected from the investigated surface, and thus, gloss values are expected to be different with materials with different surface refractive indexes (as in this study), and any changes observed in the same material should be the result of the treatment that was applied to the material surface. Following the classification provided by the ASTM D523 standard [50], metallic framework and denture base materials are classified as high-gloss materials (>70 GU [50]), while artificial teeth are classified as semi-gloss materials (10–70 GU [50]). All groups demonstrate a significant increase in surface gloss after aging, but since the study indicated that the effect of solutions on surface roughness parameters was insignificant, gloss changes cannot be explained by a smoother surface. For the Co-Cr-Mo alloy, the increase in gloss after their immersion in the solutions is also difficult to explain. It could be that all solutions had a cleansing effect on the alloy surface, disrupting the oxide layer formed on its polished surface. This hypothesis may also provide an explanation as to why HYP, ETH, and PIO have a greater effect on gloss since HYP and PIO are the solutions with the lowest pH, while ethanol is extensively used to clean metallic surfaces for fractographic analysis [59]. However, these are speculations, and therefore, further research is needed to explain the gloss increase in denture base materials in combinations within the investigated solutions.

The CIELab system was used for color measurements instead of the more complex CIEDE2000, since a similar human eye perception of color differences was found for the two formulas [60]. Following the ISO/TR 28642 requirements, a ΔE*ab value at or below 1.2 after aging depicts an excellent match (below the perceptible threshold), a value between 1.2 and 2.7 corresponds to an acceptable match, and values above 2.7 represent an unacceptable match [52]. Although these thresholds are applied for tooth-colored restorative materials, they have been extended to the rest of the materials tested in this study, as in previous studies [16,32], in the absence of respective thresholds set by international standards. Both the denture base and acrylic teeth groups showed excellent color matching after aging, and thus, their color remained unaffected by disinfection solutions. This is in accordance with previous findings for distilled water and 0.12, 0.5, 1, and 5.25% NaOCl, even for prolonged immersion times [16,32,44]. Moreover, ΔE*ab values were found below PT for artificial teeth tested in the same disinfectant solutions used in this study, even for longer immersion times [44].

Only the Co-Cr alloy showed ΔE*ab values higher than the acceptability threshold (HYP) and perceptible threshold for ETH and PIO. The groups of HYP and ETH demonstrated a significant shift in L* (towards white), matching with the high increase in gloss values, while PIO depicted a shift towards green and blue, probably due to the golden-brown color of povidone-iodine. These findings may be explained by the cleaning actions of ethanol as it is extensively used for cleaning metallic surfaces, while hydrogen peroxide and povidone-iodine are the most acidic disinfectant solutions with pH values of 6.0 and 5.7, respectively. However, this is only an assumption, and the real mechanism for these findings requires additional experimental testing. For this reason, studies are needed to investigate the long-term effect of solutions containing hydrogen peroxide like whitening solutions, cleansers, and disinfectants on Co-Cr alloys used in the fabrication of removable dentures.

It is noteworthy to mention that this study is not free from the inherent limitation of laboratory studies where the simulation of oral conditions (including the fluctuation in temperature and oral pH, saliva flow, masticatory wear, and many other factors) is impossible, and thus, the experimental findings cannot be directly projected to clinical conditions. Although the artificial aging regime substantially simulated the aging of denture base materials, in vivo aging cannot incorporate surface alterations due to instantly developed biofilm under intra-oral aging.

Although the tested disinfectants did not deteriorate the surface roughness, gloss, and color stability of materials tested, further clinical studies are required to characterize the long-term contribution on clinical performance and aging mechanisms of denture base materials.

## 5. Conclusions

Based on the findings of this in vitro study, the following conclusions were drawn:

1. The roughness of the denture material tested was not affected by the tested disinfectants.
2. The use of disinfectants had a positive effect on the gloss of all materials tested.
3. All materials tested demonstrated substantial color stability after disinfections except the Co-Cr alloy with hydrogen peroxide, and from this standpoint, the use of H_2_O_2_ should be avoided.

## Figures and Tables

**Table 1:** Concentration and pH values of disinfectant solutions used along with immersion regime (cycles × time per cycle (min)) and group codes.

Disinfectant Solutions	Concentration (wt%)	pH	Immersion Regime	Code
Distilled H_2_O		6.8	15 min ^1^	WAT
Sodium Hypochlorite NaOCl	0.1	7.5	15 × 1 min = 15 min	SOD
Hydrogen Peroxide H_2_O_2_	0.5	6.0	15 × 1 min = 15 min	HYP
Ethanol C_2_H_6_O	78	6.8	15 × 0.5 min = 7.5	ETH
Povidone iodine (C_6_H_9_I_2_NO)	1	5.7	15 × 1 min = 15 min	PIO

^1^ Note: immersion time in water was continuous.

**Table 2:** Mean values and standard deviations are shown in parentheses for all surface roughness parameters before (T0) and after (T1) artificial aging for the metallic framework (MF), denture base (DB), and artificial teeth (AF).

Metallic Framework										
Groups	Sa (nm)		Sq (nm)		Sz (nm)		Sc (nm)		Sv (nm)	
	T_0_	T_1_	T_0_	T_1_	T_0_	T_1_	T_0_	T_1_	T_0_	T_1_
WAT	144 (14)	152 (21)	190 (19)	197 (24)	3256 (1850)	2953 (860)	188 (24)	201 (35)	25 (2)	26 (2)
SOD	132 (21)	131 (20)	175 (27)	173 (20)	3256 (1237)	2652 (540)	171 (39)	171 (28)	23 (2)	24 (2)
HYP	117 (17)	118 (28)	157 (19)	161 (32)	2760 (924)	3508 (1388)	142 (30)	147 (38)	22 (1)	24 (4)
ETH	128 (25)	144 (21)	174 (33)	190 (22)	3922 (1999)	3095 (1227)	166 (42)	186 (38)	24 (2)	26 (1)
PIO	127 (16)	136 (22)	167 (20)	178 (28)	2599 (443)	2777 (399)	166 (30)	175 (32)	22 (1)	23 (2)
Denture Base										
WAT	112 (25)	94 (3)	158 (51)	172 (41)	4463 (1326)	5083 (2062)	167 (38)	130 (47)	22 (7)	24 (8)
SOD	113 (30)	100 (33)	185 (50)	162 (35)	5365 (2220)	5468 (2048)	160 (45)	145 (49)	26 (6)	23 (6)
HYP	106 (57)	106 (37)	184 (97)	170 (62)	6888 (5423)	5486 (3928)	153 (84)	147 (55)	25 (15)	25 (8)
ETH	118 (53)	122 (44)	185 (79)	191 (60)	6618 (3042)	5908 (2089)	169 (79)	175 (68)	26 (12)	28 (9)
PIO	121 (69)	140 (97)	192 (90)	176 (45)	7937 (3906)	7132 (4523)	174 (95)	193 (133)	23 (8)	27 (7)
Artificial Teeth										
WAT	48 (28)	73 (17)	75 (43)	116 (33)	2322 (650)	2882 (680)	74 (42)	109 (26)	6 (3)	9 (1)
SOD	70 (11)	79 (13)	109 (20)	120 (20)	3086 (1211)	3590 (1438)	108 (22)	122 (26)	10 (1)	11 (1)
HYP	70 (10)	77 (11)	105 (16)	118 (19)	2383 (427)	2325 (453)	107 (17)	118 (20)	9 (1)	11 (1)
ETH	68 (18)	72 (9)	109 (30)	106 (14)	2483 (785)	2582 (692)	100 (29)	106 (14)	10 (2)	11 (1)
PIO	75 (10)	80 (14)	115 (14)	118 (21)	3137 (955)	2608 (638)	115 (19)	122 (24)	10 (1)	12 (2)

No statistically significant differences were identified between T0 and T1 for all materials tested (*p* > 0.05).

**Table 3:** Mean values and standard deviations are shown in parentheses, and the median and 25% and 75% percentiles are shown in brackets for gloss values (gloss units) of the Co-Cr alloy, denture base material, and artificial teeth before and after immersion cycles, along with *p* values. ΔG values with the median and 25% and 75% percentiles are shown in brackets.

Metallic Framework				
Group	T_0_	T_1_	*p*	ΔG
WAT	230 (15)	233 (14)	<0.001	2.6 [1.4–4.8] ^A^
SOD	297 [259–309]	308 [301–327]	0.002	14.1 [3.8–44.5] ^AB^
HYP	260 [239–287]	306 [285–327]	0.002	39.7 [28.0–67.8] ^B^
ETH	252 (44)	296 (38)	<0.001	37.0 [29.9–62.5] ^B^
PIO	280 (41)	307 (46)	0.003	26.7 [4.9–43.2] ^AB^
Denture Base				
WAT	95 (12)	103 (7)	0.004	7.1 [2.3–11.4] ^A^
SOD	97 (6)	105 (10)	0.009	9.4 [1.1–13.7] ^A^
HYP	96 [79–104]	103 [86–109]	0.002	2.4 [0.7–9.2] ^A^
ETH	86 [82–101]	89 [84–110]	0.002	1.2 [0.5–5.4] ^A^
PIO	93 [78–99]	97 [81–107]	0.002	2.6 [1.0–6.9] ^A^
Artificial Teeth				
WAT	12 (2)	26 (5)	<0.001	13.9 (4.8) ^AB^
SOD	10 (1)	28 (5)	<0.001	17.9 (6.4) ^B^
HYP	10 (2)	20 (3)	<0.001	10.0 (4.1) ^A^
ETH	12 (1)	22 (4)	<0.001	9.8 (4.5) ^A^
PIO	11 (1)	22 (5)	<0.001	10.8 (6.1) ^A^

Significant differences between T0 and T1 are indicated by *p* values (horizontal comparison). Different capital letters indicate significant differences among ΔG values (vertical comparison, *p* < 0.05, right column).

## Data Availability

The original contributions presented in the study are included in the article, further inquiries can be directed to the corresponding author.

## Supplementary Materials

The following supporting information can be downloaded at https://www.mdpi.com/article/10.3390/jfb16120446/s1, Supplementary Data.xls. All raw data for surface roughness, gloss, and color are available in an Excel file.
